# Exploring Children’s Physical Activity Behaviours According to Location: A Mixed-Methods Case Study

**DOI:** 10.3390/sports7110240

**Published:** 2019-11-18

**Authors:** Irfan Khawaja, Lorayne Woodfield, Peter Collins, Adam Benkwitz, Alan Nevill

**Affiliations:** 1 Department of Sport and Exercise, Birmingham City University, Birmingham B15 3TN, UK; 2 Department of Social Science, Sport and Business, Newman University, Birmingham B32 3NT, UK; L.A.Woodfield@staff.newman.ac.uk (L.W.); A.Benkwitz@staff.newman.ac.uk (A.B.); 3 Faculty of Education, Health and Wellbeing, University of Wolverhampton, Wolverhampton WS1 3BD, UK; Peter.Collins@wlv.ac.uk (P.C.); a.m.nevill@wlv.ac.uk (A.N.)

**Keywords:** global positioning system, physical activity, location, mixed methods

## Abstract

The school environment is ideally placed to facilitate physical activity (PA) with numerous windows of opportunity from break and lunch times, to lesson times and extracurricular clubs. However, little is known about how children interact with the school environment to engage in PA and the other locations they visit daily, including time spent outside of the school environment i.e., evening and weekend locations. Moreover, there has been little research incorporating a mixed-methods approach that captures children’s voices alongside objectively tracking children’s PA patterns. The aim of this study was to explore children’s PA behaviours according to different locations. Sixty children (29 boys, 31 girls)—35 key stage 2 (aged 9–11) and 25 key stage 3 (aged 11–13)—wore an integrated global positioning systems (GPS) and heart rate (HR) monitor over four consecutive days. A subsample of children (n = 32) were invited to take part in one of six focus groups to further explore PA behaviours and identify barriers and facilitators to PA. Children also completed a PA diary. The KS2 children spent significantly more time outdoors than KS3 children (*p* = 0.009). Boys engaged in more light PA (LPA) when on foot and in school, compared with girls (*p* = *0.003*). KS3 children engaged in significantly more moderate PA (MPA) at school than KS2 children (*p* = 0.006). Focus groups revealed fun, enjoyment, friends, and family to be associated with PA, and technology, costs, and weather to be barriers to PA. This mixed methodological study highlights differences in the PA patterns and perceptions of children according to age and gender. Future studies should utilize a multi-method approach to gain a greater insight into children’s PA patterns and inform future health policies that differentiate among a range of demographic groups of children.

## 1. Introduction

Regular physical activity (PA) is associated with wide-ranging health benefits [1,2], and the benefits of PA during childhood have been widely reported [3,4,5] with current PA guidelines for children and youth recommending that they accumulate at least 60 min of daily moderate–vigorous physical activity (MVPA), including a variety of types and intensities of PA across the week to develop movement skills, muscular fitness, and bone strength [6,7]. The key benefits of engaging in regular PA have been previously outlined and include: improved cardiovascular health, maintenance of a healthy weight, improved bone health [7,8], and improved self-efficacy [9]. Children’s habitual physical activities such as playing outdoors, and walking and cycling for transport provide opportunities to be physically active throughout the day [10]. In support of this, instilling habitual PA in young children is crucial, as PA behaviors tend to track from childhood to adolescence and adulthood [11].

With the current generation of children reporting lower levels of PA, concerns are being raised on the impact this will have on their growth, lifestyle, and physical and mental development [9]. Research amongst children is particularly warranted because of the low numbers of children who are meeting the daily recommended levels for PA [7,12,13,14]. UK figures show that only 23% of boys and 20% of girls meet daily recommended PA levels [15]. Furthermore, 14% of boys and 10% of girls in the West Midlands, England specifically meet the PA guidelines, which nationally, is the lowest for both genders [16].

Reducing this specific decline in age-related PA behaviours has been identified as an important health priority, as inactivity tends to track into adulthood [17]. Research suggests that MVPA drops by up to 7% per year between the ages of 9 and 15 years, so that by the age of 15, the majority of young people no longer meet the recommended daily amount of activity [18]. Therefore, the transition from primary to secondary school education (i.e., between key stage 2 and key stage 3) is as a key area of interest when monitoring PA among children in the UK.

PA behaviour can be influenced by the surrounding environment, and adapting this environment could result in improved health-related behaviours [19]. Time spent outdoors is consistently associated with higher daily PA in children; however, parents often limit children’s levels of outdoor play in response to concerns about safety [20]. Restricting children to the home environment has been linked with lower levels of active transport and MVPA, greater levels of sedentary time in children [21], and declining amounts of children’s PA [12]. Therefore, there is a need to explore children’s independent mobility and the freedom to play outdoors in relation to PA and the built environment [12].

Global positioning systems (GPS) is a tool that is used to measure location and is increasingly popular in health research, with many studies combining GPS with other measures such as heart rate (HR) monitors and accelerometers [22,23,24,25,26,27]. Combining GPS with heart rate monitors provides an objective measure to determine the intensity of PA according to different locations [22,23,28,29]. GPS monitoring shows promise as a method to improve understanding of how the built environment influences PA behaviours by allowing activity to be quantified in a range of physical contexts [30]. 

Identifying barriers and facilitators to PA is key in order to understand children’s PA, but it is also important to inform future interventions aiming to increase PA in this group [31]. Previous research has used focus groups in conjunction with measures of PA [26], and a thematic approach to analyse the data has been modelled [32]. This allows the researcher to gain a deeper insight into children’s thoughts about PA and their PA experiences. This type of information can add a greater meaning and context to any quantitative data collected [33].

Current literature indicates that there are a multitude of factors that affect levels of PA. However, there appears to be a lack of studies that have applied a mixed-methods approach to objectively measure children’s PA behaviours according to location, specifically using GPS, and to establish the reasons behind their PA participation [34]. This study aims to use a mixed methods approach implementing GPS, HR monitors, focus groups, and PA diaries to explore children’s PA behaviours according to location, and how this differs according to gender and key stage. Therefore, this study will aim to address the following research question: Do PA behaviours and locations for PA differ according to gender and age?

## 2. Materials and Methods

### 2.1. Participants

School children (9–13 years) from one middle school in the West Midlands, United Kingdom were invited to participate in this study (school data: 560 total children, 17% from ethnic minority groups, 21% free school meal eligibility, which is greater than the current 13.6% national average [35]). Following institutional ethical approval, informed consent was gained from the head teacher of the school, parents/guardians, and from the children. After promoting the investigation within the school, an opportunity sample of 119 children aged 9–13 years (21% of the total school population) consented to participate in the study (see Table 1).

### 2.2. Procedures

Details of how to use and wear GPS and HR equipment were explained to children, which included specifics on wearing the heart rate monitor chest strap, and how to operate and recharge the GPS watch. Instructions were also provided on when not to wear the equipment (i.e., water-based activity, contact sports, etc.). Children wore GPS watches (Garmin Forerunner 305, Garmin Ltd., Olathe, KS, USA) and synchronised HR monitors daily over a four-day period, which has been supported by previous literature [24]. In the context of this study, the four-day period enabled two weekdays to be compared with two weekend days (Thursday–Sunday: as soon as children woke up until going to bed), which provided a reflective indication of daily PA.

Children also completed a modified version of an original ‘PA Diary’ [36]. The PA diary asked children to complete four sections, including type of activity, intensity of activity, time spent on activity, and whether the GPS monitor was worn [37]. As GPS and HR devices were not worn in water-based activities (i.e., swimming, bathing, etc.) and some activities required the removal of the devices for health and safety reasons, the PA diary would provide a record of this. Using a PA diary alongside other measures provides an opportunity for data to be cross-referenced [38].

A random stratified sample (according to gender and key stage) of 32 children who provided a minimum of one hour of combined GPS and HR data over the four days were invited to take part in one of six focus groups. Focus groups have been identified as being a popular method of qualitative data collection with children [34]. Focus groups were facilitated by the lead researcher, and took place during the school lunch time, were based in a school classroom, and were recorded using an mp3 audio recorder.

The social–ecological model was used as a framework to underpin questions/topics for discussion in the focus groups [39]. Focus group questions were constructed using a deductive approach, as an indication of PA levels had been provided from the quantitative data. The objective of focus groups was to explore the children’s perspectives on their motives and locations for PA, and to identify the types of PA that were undertaken. Children’s responses from focus groups were also used to identify facilitators and barriers to PA participation.

### 2.3. Data Processing and Statistical Analysis

GPS and HR data were downloaded via the Garmin Connect website (Garmin Ltd., Olathe, KS, USA) which individually stored children’s data. GPS Utility software was used to extract the raw GPS and HR-matched data. The GPS location points were manually entered into Google Maps to establish location. An index of locations was categorised as follows based on the visited locations in which children spent time: ‘Home’, ‘School’, ‘Other indoor location’, ‘Motorised transport’, ‘On foot’, ‘Outdoors’, and ‘Outside’ (combining ‘on foot’ and ‘outdoors’). Then, data were cleaned by analysing each child’s profile of data and removing time fields where GPS and HR data were missing. Following the cleaning of the GPS and HR data, 60 out of the original 119 children’s profiles met the one-hour minimum data inclusion criteria, and were eligible for analysis (Boys = 29, Girls = 31; KS2 = 35, KS3 = 25), and 80 children returned PA diaries (Boys = 32, Girls = 48, KS2 = 41, KS3 = 39), which was inclusive of the 60 meeting GPS and HR inclusion criteria. PA diaries were useful in providing greater context to the PA data from the HR monitors and location data from the GPS monitors. A total of 32 children who provided a minimum of one hour combined GPS and HR data were invited to attend one of six focus groups (Boys = 16, Girls = 16; KS2 = 16, KS3 = 16).

PA intensity thresholds were established by calculating each child’s heart-rate reserve. The heart-rate reserve is the difference between a children’s resting and maximum HR [40] recorded using the integrated heart-rate monitor over the observation period. Then, this was used to establish children’s HR intensities. In line with previous guidelines, the Karvonen method of identifying percentage training intensities was applied [41]. The PA intensity percentage thresholds of heart-rate reserve [40] were labelled as follows: 0–29%—Sedentary, 30–49%—Light, 50–75%—Moderate, above 75%—Vigorous.

A range of statistical procedures were carried out to explore GPS and HR data using 95% confidence intervals throughout. Results from a Priori sample size calculation exploring MVPA exceeded the sample, which limited statistically significant findings (Min total sample size: n = 128, Min sample size per group 64, Desired power = 0.8, Cohen’s *d* = 0.45, *p* = 0.09). Univariate ANOVA tests were used to explore data for key stage and gender interactions between MVPA and time spent in different locations. Furthermore, Pearson’s product moment correlations were used to establish relationships between daily MVPA and location. Independent samples t-tests were used to explore gender and key stage differences, with effect sizes calculated for statistically significant findings. A two-tailed significance value of *p* < 0.05 was considered as being statistically significant in all statistical analyses. Descriptive statistics were provided for the analyses, including mean MVPA, sedentary behaviour (SB), light PA (LPA), moderate PA (MPA), and vigorous PA (VPA) according to different locations, and reports of meeting recommended daily PA guidelines. SPSS version 25 (SPSS Inc., Chicago, IL, USA) was used for all statistical analyses.

Focus group data were transcribed, and a thematic analysis was undertaken to explore and develop an understanding of the PA experiences of the children [42,43]. This enabled further insights into the potential reasons/motives for PA, which included identifying barriers and facilitators to children’s PA [26]. 

## 3. Results

### 3.1. Children’s PA Levels

Analysis was conducted on the datasets of the 60 children meeting the inclusion criteria. Descriptive statistics for time spent in different HR intensities (including all children meeting the inclusion criteria, n = 60), and children meeting PA guidelines (n = 31) are provided in Table 2. Thirty-three children (52%) met the daily 60-min PA guidelines, and 29 children (48%) did not meet the guidelines [44].

Analyses of children’s’ PA behaviours revealed one statistically significant result, where KS3 boys were more sedentary than KS3 girls (KS3 boys = 57.1%, KS3 girls = 37.2%; *p* = 0.016; KS3 boys = 260.9 min, KS3 girls = 153 min; [CI = 22.4:193.5], large effect size: *d* = 1.04). Therefore, the following results are based on descriptives.

When exploring daily mean MVPA minutes and percentage of MVPA time, children in KS3 spent 26.9 min (2.3%) longer in MVPA than KS2 children. Additionally, girls demonstrated 38.4 min more mean daily MVPA (7.1%) than boys. After investigating gender differences according to key stages, MVPA was similar between KS2 boys and girls; however, KS3 girls engaged in more MVPA than KS3 boys (KS3 boys = 9.6%, KS3 girls = 24%).

The percentage of time spent in SB was similar across genders and key stages, and LPA was similar between key stages (KS2 = 36.5%, KS3 = 36.4%). However, boys spent a higher proportion of their time in LPA (39.6%) compared to girls (33.6%). Further gender differences revealed girls to engage in more MPA (17.9%) than boys (11.7%). MPA behaviour between key stages showed older children to engage in more MPA time than younger children (KS2 = 14%, KS3 = 16.2%). VPA was similar across both genders and key stages (Boys = 2.8%, Girls = 3.1%, KS2 = 2.9%, KS3 = 3%).

When exploring data for gender differences according to each key stage, KS2 girls were more sedentary than KS2 boys (KS2 boys = 40.5%, KS2 girls = 53.9%). KS2 boys engaged in a higher percentage of LPA compared with KS2 girls (KS2 boys = 42.6%, KS2 girls = 28.4%); however, this pattern was reversed at KS3: KS3 girls engaged in more LPA than KS3 boys (KS3 boys = 32.9%, KS3 girls = 38.4%). MPA was similar between KS2 boys and girls; however, KS3 girls engaged in more MPA than KS3 boys (KS3 boys = 7.3%, KS3 girls = 21.2%). VPA was similar between boys and girls in KS2, and boys and girls in KS3 (KS2 boys = 2.8%, KS2 girls = 3.1%, KS3 boys = 2.7%, KS3 girls = 3.2%). 

Investigation into any potential impact device wear time had on MVPA was carried out. Results from this study revealed a weak relationship when exploring weekday 1 and 2 (Thursday and Friday), and weekend day 1 (Saturday) total wear time with MVPA (weekday 1: *r* = 0.354, *p* < 0.01; weekday 2: *r* = 0.439, *p* < 0.01; weekend day 1: *r* = 0.550, *p* < 0.01). Weekend day 2 (Sunday) was the only day to report a stronger relationship between device wear time and MVPA (*r* = 0.848, *p* < 0.01); however, this was due to a reduced number of children engaging in MVPA (n = 4) on this specific day, and therefore cannot be used as a generalisable finding.

To further support this, mean wear time (over the four-day period) and MVPA time reported weak correlations (*r* = 0.322, *p* < 0.05). This indicates that children who wore devices for greater durations did not necessarily report greater amounts of MVPA. Additionally, similar findings were reported when exploring wear time and maximum HR (*r* = 0.356, *p* < 0.01). This suggests that a higher maximum HR was not associated with children who reported greater device wear time.

PA diaries revealed that four children removed monitoring equipment due to swimming activities. This activity was described as ‘tiring’, ‘very tiring’, or ‘tough’. There were some activities such as football, gymnastics, and taekwondo where equipment was also removed for health and safety reasons. These activities were described as ‘hard’ or ‘tiring’. Other general reasons why equipment was removed involved completing household chores, caring for a pet, or travelling on family holidays.

### 3.2. Time Spent in the Surrounding Environment.

When exploring percentage of time spent according to location, girls spent significantly more time in motorised transport than boys (*p =* 0.21; girls = 17.7% (±17.6), boys = 8.3% (±12.4); (CI = −17.26: 1.43), medium effect size: *d* = 0.61), and boys also spent significantly more time in school than girls (*p =* 0.006; boys = 41.4 (±22.7), girls = 23.4 (±26.4); (CI = 5.29:30.83), medium effect size: *d* = 0.73). KS3 children spent significantly more time at home compared with their KS2 counterparts (*p =* 0.010; KS3 = 41.6 (±27.7), KS2 = 24.8 (±21.2); (CI = −29.38:4.12), medium effect size: *d* = 0.68); however, those in KS2 spent significantly more time in motorised transport than KS3 children (*p* = 0.046; KS2 = 16.6 (±18.4), KS3 = 8.4 (±10); (CI = 0.16:16.38), medium effect size: *d* = 0.56). KS2 children also spent significantly more time outdoors than KS3 children (*p* = 0.009; KS2 = 19.1 (±12.7), KS3 = 10 (±13.1); (CI = 2.35: 15.84), medium effect size: *d* = 0.71), and a similar statistically significant finding was found when analysing time spent ‘outside’, with KS2 children spending more time ‘outside’ than their KS3 counterparts (*p* = 0.023; KS2 = 19.4 (±13.2), KS3 = 12.4 (±8.8); (CI = 1:13.14), medium effect size: *d* = 0.63). When investigating gender within each key stage, KS2 girls spent significantly more time in motorised transport than KS2 boys (*p* = 0.048; KS2 girls = 23.7 (±21.6), KS2 boys = 11.3 (±13.9); (CI = −24.54: −0.14), medium effect size: *d* = 0.68). This trend was repeated by KS3 children, as KS3 girls also engaged in significantly more time in motorised transport than KS3 boys (*p* = 0.010, KS3 girls = 12.1 (±10.8), KS3 boys = 1.7 (±2.4); (CI = −17.99: −2.75), large effect size: *d* = 1.32). KS2 boys also spent significantly more time at school than KS2 girls (*p* = 0.037, KS2 boys = 40.2 (±23.8), KS2 girls = 22.5 (± 23.8); (CI = 1.1:34.12), medium effect size: *d* = 0.74).

**Figure 1:**
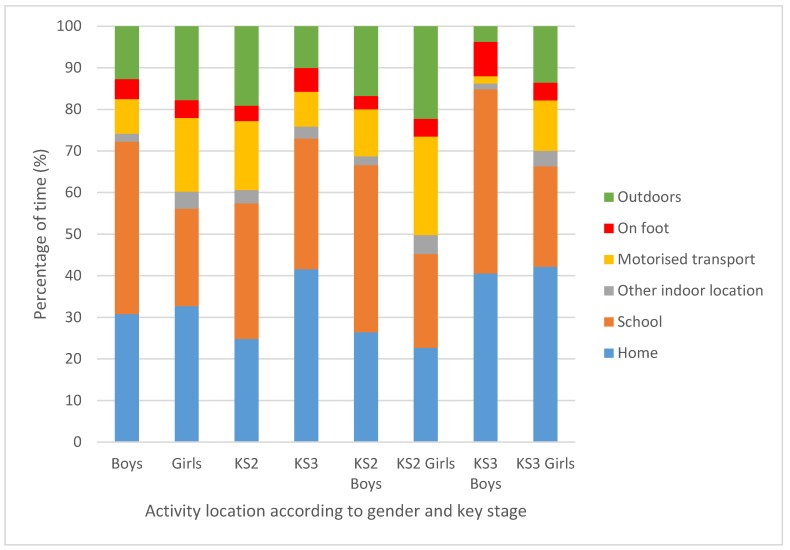
Mean percentage of time spent in different locations according to gender, key stage, and gender within key stage.

Descriptives for percentage time spent in different locations according to gender, key stage, and gender within each key stage are presented in Table 3 and Figure 1.

### 3.3. PA Intensities According to Location

**Figure 2:**
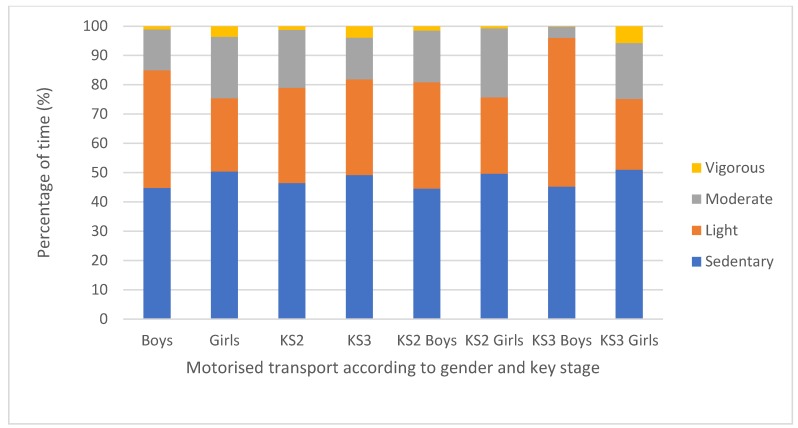
Mean percentage of time in each HR intensity spent in motorised transport according to gender, key stage, and gender within key stage.

When investigating HR according to location, a paired-samples t-test revealed no statistically significant differences between MVPA at school and outside of school. Children spent 9.7% of their time out of school in MVPA, which was 2.5% more than that in school. An independent t-test revealed significant gender differences, with boys engaging in significantly more LPA in motorised transport compared with girls (*p* = 0.026; Boys = 40.2% ± 27.1, Girls = 25% ± 20.2; (CI = 1.87:28.51), medium effect size: *d* = 0.64) (see Figure 2). A finding that was approaching significance (*p* = 0.05) was revealed in the school environment with KS3 children engaging in more MPA than KS2 children (KS2 = 12.2% ± 9.4, KS3 = 21.1% ± 19.9).

**Figure 3:**
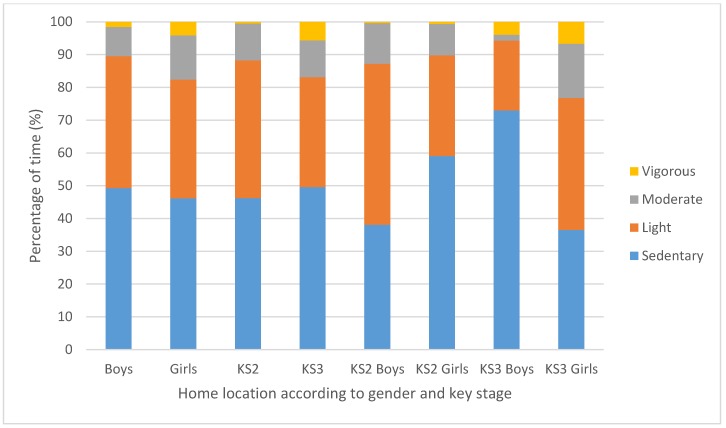
Mean percentage of time spent in each HR intensity at home according to gender, key stage and gender within key stage.

Gender by key stage results showed KS3 boys to engage in significantly more LPA than KS3 girls when in motorised transport (*p* = 0.01; KS3 boys = 50.8% ± 27.7, KS3 girls = 24.2% ± 16.2; (CI = 7.16:46.01), large effect size: *d* = 1.17, see Figure 2). Additionally, KS3 boys also engaged in significantly more SB than KS3 girls when at home (*p* = 0.014; KS3 boys = 73% ± 29.4, KS3 girls = 36.5% ± 34.4; (CI = 8.27:64.68), large effect size: *d* = 1.14, see Figure 3). Another finding that was approaching significance (*p* = 0.05) was KS2 boys engaging in more VPA than KS2 girls when at school (KS2 boys = 3.7% ± 3.3, KS2 girls = 1.3% ± 2).

### 3.4. PA Barriers and Facilitators

During focus groups, children described PA behaviours and how these changed according to school term and weather. Furthermore, focus groups also allowed children to discuss different reasons, barriers, and facilitators for PA behaviour. This provided greater context and meaning, and the researcher was able to gain a deeper, more accurate articulation of children’s thoughts, which further supported the quantitative data. 

Thematic analysis enabled children’s PA behaviour to be explored according to the different locations visited, which was provided by HR and GPS data. The components of the social–ecological model [39] provided first-order themes for the focus group transcripts to be analysed. Then, second-order themes could be categorised according to barriers to PA or facilitators for PA. Boys and girls in both key stages reported similar barriers and facilitators within their discussions. Themes and raw data that contributed to focus groups are provided in Table 4.

The qualitative results suggested a range of influential factors that children felt affected their PA, both encouraging PA, and barriers towards PA (see Table 4). Some of the more meaningful comments from the data collection are presented below.

#### 3.4.1. Barriers to PA (Categorised According to Relevant Social–Ecological Component)

##### ‘*Individual*’ Component

Children identified the cost of equipment as a reason that prevented them from PA participation: *“Maybe lower the price a little bit so that people don’t have to wait loads to save up.”* This would indicate that children’s PA was dependent upon personal family circumstances and financial costs associated with PA. However, the school environment that provided PA opportunities at no cost was also discussed: *“Like do a survey to see what they would want to do instead of like saying we’re going to do hockey today because some people might not like it”*; *“People might not like what the variety is.”* Therefore, children felt that the lack of variety of PA available consequently meant that there was limited uptake in PA participation.

##### ‘*Social Environment*’ Component

Family commitments and responsibilities were highlighted as a potential barrier: *“Sometimes some of the people might have other plans and like they might have problems with their family and might have to look after their family and it might stops [sic] you having their social time with their friends and going out.”*

Location was reported as an influencing factor towards PA: *“I go up to the college car park because it’s big and loads of my friends just play there.”* This location was not specifically designed to promote PA, but children saw this location as an opportunity to increase PA.

##### ‘*Other*’ Component

Children also felt that the weather restricted the amount of PA that could be completed: *“… if it’s in the winter, some people don’t have motivation because it’s quite cold and dark, and if it’s muddy.”* Therefore, this would suggest that during winter seasons specifically, children preferred alternative locations for PA. Finally, children highlighted technology as a factor that was a barrier to PA: “*Consoles are the most distracting thing you can have”*; *“If we didn’t have an Xbox, we would probably still go out.”*

#### 3.4.2. Facilitators for PA (Categorised According to Relevant Social–Ecological Component)

##### ‘*Individual*’ Component

Children highlighted the ‘fun’ element of PA clubs as this would encourage PA promotion: *“a club that’s fun and active, and people would like to go to it and it’s not too far.”* This also voiced how children enjoy the ‘active’ nature of PA clubs, which may in turn contribute towards the enjoyment factor.

##### ‘*Physical Environment*’ Component

Location is once again mentioned, meaning that ease of access would further encourage PA. The social aspect of PA was identified as being a key factor affecting PA: *“We kind of meet up because that’s where we all have our talk and mainly have our discussions and have more time there because it’s the closest place for us to meet up.”* This suggests that children were more likely to engage in PA with peers and in locations that were central and accessible. A similar pattern was found regarding family: *“I do runs with my dad, but I don’t do the runs, I go on my bike and I just go sometimes to the gym with him.”*

##### ‘*Policy*’ Component

When discussing how PA could be promoted, children identified the importance of consulting the ‘student voice’: *“You can ask what they like the most, and start clubs and get them to come”.* Children also felt that a PA-related reward system would promote further PA: *“… in periods of time when who does the most exercise you win a prize, so you’re kind of pushing the student to work for the prize.”* This proposition would encourage a new school policy on rewarding PA behaviours.

##### ‘*Other*’ Component

Finally, children identified a ‘solution-focussed’ approach regarding technology: *“You could reduce your time down on a tablet or computer so that you’re not always on it”*; *“However long you do on a physical activity, you get half that time on technology or something like that”*.

## 4. Discussion

This study aimed to use a mixed-methods approach to explore children’s PA behaviours according to location. Fifty-two percent of boys and 48% of girls in the study met the 60-min minimum MVPA guidelines for the UK [7], which is a greater percent than national statistics where 23% of boys and 20% girls met recommended guidelines [15]. Although there was a greater number of boys meeting the MVPA guidelines, the results reported from this study indicate girls to spend a higher proportion of their time in MVPA (20.9%), compared with boys (13.8%). This suggests that despite fewer girls meeting the MVPA guidelines, those girls who did engage in MVPA engaged in greater MVPA time, which consequently increased girls’ mean MVPA. However, this trend is in direct contrast to previous literature [7,15,45,46,47], which identified boys to be more active than girls. More specifically, girls report smaller amounts of time spent in LPA, MPA, and VPA in previous research [15,45,46]. Further UK research concludes that boys were more likely to be physically active [48]. Most PA opportunities in primary schools (which most KS2 children attend) are mixed-gender. When these children move into secondary schools (for KS3), they traditionally receive PA opportunities (PE and extracurricular provision) for boys and girls separately. However, in the participating school, a middle school, both KS2 and KS3 children had mixed-gender provision. Therefore, the mixed-gender provision for KS2 and KS3 at the participating school provides a possible explanation for the findings of the current study (i.e., girls meeting PA guidelines, and participating in more MVPA than boys). 

Investigations exploring PA behaviours across transitional periods between age groups has revealed a reduction in PA with age [18,49,50,51]. However, findings from the current study revealed that 58% of KS2 children and 42% of KS3 children met the recommended daily PA guidelines [7], and KS3 children spent a higher proportion of their time in MVPA (18.8%) compared with KS2 children (16.5%). These findings differ from the age-related decline in PA discussed in previous literature [18,49,50,51]. This may again be due to the environment and education structure of the participating school in this study. The majority of children in the UK change schools when transitioning from KS2 to KS3 (i.e., move from primary to secondary school), whereas the participating school in this study was a middle school, where children stayed at the same school for their transition from KS2 to KS3. Therefore, the environment was consistent, potentially leading to consistent activity behaviours between KS2 and KS3. This is in contrast to published literature in the UK, which has identified a decline in PA between KS2 and KS3 [18,50]. Additionally, this particular school had a designated gym that was not used for other purposes i.e., lunch hall, school assembly, etc. The school also benefited from having a large playing field that children could make use of during warmer, dryer months of the year, and encouraged greater PA. These facilities may have also contributed towards these PA findings.

Evidence indicates that PA encourages association of PA with enjoyment [52,53]. Within focus groups, when discussing reasons for PA participation, students stated: *“It just makes me happy and [I] want to do it all the time.”* This would suggest that children acknowledge the enjoyment of PA, and this was a reason for continued participation. In addition to this, the children may have had a degree of input on the PA the school offered, and research suggests that children’s individual preferences on choice of PA leads to greater enjoyment and participation [54].

The school has been identified as a location that promotes PA [55,56,57]. Previous research has identified key windows during the day to contribute to school-based PA, as break time and other extracurricular periods provide opportunities for PA and fitness [58]. However, within this study, time spent in school (including academic, break, and lunch times) resulted in 7.3% MVPA, compared with 9.7% MVPA outside of school. Therefore, it could be suggested that there may be factors outside of the school environment that encourage greater PA behaviours, and barriers within school that may inhibit PA behaviours e.g., indoor sedentary lessons. In order to clarify this further, daily minutes of MVPA for the two environments were explored. The pattern of results showed children to spend 32.6 min of daily MVPA within school, and 45.9 min of daily MVPA outside of school. 

Current literature indicates that environmental attributes such as place of residence and the accessibility of recreational facilities influence levels of PA [59]. Although time spent outdoors is consistently associated with higher daily PA in children, parents often limit children’s levels of outdoor play in response to concerns about safety [19,20]. This was highlighted within focus groups, as when children were discussing locations for PA, it was proposed: *“have more time there because it’s the closest place for us to meet up”.* This study revealed that girls spent significantly more time in motorised transport than boys, and KS2 children spent significantly more time in motorised transport than KS3 children. Boys and KS3 children also spent more time on foot (time spent walking/commuting) compared with girls and KS2 children. This would support safety concerns and environmental patterns previously reported in the literature suggesting that boys and older children have greater freedom than girls and younger children [19,20].

While older children in the current study appear to have more freedom, those in key stage 3 spent more time within the home and less time outdoors in comparison with key stage 2 children. Access to parks and open green spaces have been identified as being destinations for PA; however, studies show that these locations have been underused [60,61,62,63,64]. The lack of time that KS3 children in the current study spent visiting green spaces and public parks supports these previous findings [60,61,62,63]. KS2 children spent significantly more time outside (which combined outdoor time and time spent on foot) than KS3 children, suggesting that despite KS3 children being more likely to spend time on foot (walking/commuting), KS2 children were more likely to spend time outdoors, perhaps on returning from school and on weekends. Focus group findings provided one explanation for the greater amount of time at home and less time outdoors when a KS3 child stated one barrier to PA: *“If I have too much homework, exams and stuff.”* However, KS3 children also stated that children *“might have to look after their family and it might stops [sic] you having their social time with their friends and going out.”* This also suggests that KS3 children may have additional family responsibilities that mean they are less likely to engage in outdoor PA and may be confined to the home.

When investigating the school environment, boys spent significantly more time in school than girls; however, girls engaged in greater school MVPA than boys (boys = 16.9%, girls = 22.7%). KS3 children spent more of their school time in MVPA (25.3%) than KS2 children (15.1%), which is in contrast to previous literature [18,49,50]. This may be due to the school providing greater opportunities for KS3 children to engage in PA compared with children in KS2, or potential barriers to PA for KS2 within the school environment. Findings from focus groups support this as a KS2 child stated: *“People might not like what the variety is.”* Further investigation found that PA clubs that were currently being provided were dictated by older children’s preferences; therefore, there was a need to consult younger children in KS2.

Focus group data showed key stage differences relating to the use of the outside environment, as when describing outside locations, a KS2 child stated: *“It’s a chance to meet up with friends.”* Whereas a KS3 child stated: *“… because that’s where the club [is], it’s what I do and I’m a part of that team.”* This indicates how the outside environment is contextualised according to two different purposes, with a KS3 perspective referring to a PA club/team, and KS2 referring to more social aspects. 

Motorised transport was further investigated as this revealed significant gender differences, with boys engaging in significantly more LPA compared with girls (Boys = 40.2% ± 27.1, Girls = 25% ± 20.2), and there were also elevated levels of MVPA (see Figure 2). These findings were due to children attending school-based PA i.e., extracurricular clubs, and then being collected by parents/guardians by motorised transport. School policy indicated that children did not have to change out of their PE kit at the end of extracurricular clubs, therefore, children could collect belongings and meet parents/guardians. Consequently, children’s heart rates were still elevated whilst travelling in motorised transport. Future research exploring HR according to motorised transport may wish to have a standardised ‘break’ period before taking HR readings to allow for a more reflective HR whilst in motorised transport.

PA diaries provided an insight and a greater context into the different types of PA behaviours in which children engaged. Eighty out of the original 119 children returned PA diaries (Boys = 32, Girls = 48, KS2 = 41, KS3 = 39). These were of interest when investigating times where children indicated they were physically working hard, but didn’t wear the HR equipment, or no connectivity was reported due to movement of the HR sensors. The PA diaries in these instances meant that the researcher was provided with the self-reported PA of the children. Previous literature supports the use of PA diaries alongside other measures as it enables for data to be cross-referenced or collected when another source of data collection fails [38]. Examples of where children removed GPS and HR devices were found to be in water-based activities (i.e., swimming, bathing, etc.). Health and safety factors also meant that devices were removed during football, skating, gymnastics, taekwondo, and wrestling activities. Therefore, it acknowledged that PA could be undermeasured using GPS and integrated heart rate data, without a waterproof or unobtrusive device. Examples of which were when a KS2 boy removed equipment for swimming activities lasting 1.5 h, and described the activity as ‘very tiring’, and a KS3 girl removing equipment for gymnastics activities for two hours and described the activity as ‘hard’. In the context of this study’s findings, it is suggested that more boys than girls removed chest straps/watches (due to the nature of activities undertaken), which could provide a potential explanation for why girls in the current study took part in more MVPA than boys, particularly as the majority of previous literature points to girls being less active than boys [15,46,65].

The use of focus groups to explore PA behaviours in this study supports previous literature [66,67]. Children were influenced by a range of factors that both facilitated and limited opportunities to take part in PA. The children highlighted that: *“They might not like it, so they might not want to do it”* and *“… if they don’t have like the right equipment to do it.”* Despite factors such as fun, enjoyment, and wanting to keep fit and healthy as being identified as motives for PA participation on an individual level, children identified time, poor diet/food intake, and the cost of equipment as restrictions to PA participation [66,67].

The social environment surrounding the children was also identified as being a facilitator to taking part in PA. PA was often associated with a chance to ‘catch up’ or socialise with friends in a fun environment. Children stated: *“It’s a chance to meet up with friends.”* This is in accordance with previous literature, which indicates that the social aspect of PA induces peer support [67]. This was seen as a positive factor that children felt encouraged them to attend different PA clubs, inside and outside of the school environment. The family was also highlighted as encouraging PA behaviours: *“I do runs with my dad, I go on my bike.”* Therefore, within this context, both family and friends were found to promote positive PA behaviours, and this supports previously published literature [68,69]. 

The physical environment has been described as having a direct association with children’s participation in PA, and this has been supported by previous research [23]. In the current study, children identified that a PA-supporting surrounding environment positively encouraged them to take part in PA. This included associations with local parks, lakes, hills, and simple open spaces: *“I go up to the college car park because it’s big and loads of my friends just play there.”* Themes from previously published literature were evidenced in this study with the proximity and location of environments highlighted, describing that they visited such areas because they were the closest for friends to meet up, and also that there were no costs involved [67].

Other responses from children included the weather being a factor that either supported or limited PA participation. Some children described poor and ‘muddy’ conditions as a barrier to participating in regular PA, which prompted a greater demand for indoor PA clubs: *“If it’s in the winter, some people don’t have motivation because it’s quite cold and dark, and if it’s muddy.”* This is a common theme within the literature, as many studies have reported children to be more active in warmer weather, which also allows for greater outdoor PA [70,71]. However, a strong theme emerged from the focus groups relating to the use of technology as a major hindrance to PA participation. Despite current research indicating that technology can be used as a positive way to monitor and promote a PA lifestyle [66,69], children within this study often reported technology as a barrier towards PA: *“When you’ve got a phone or like a console, you don’t think as much about getting active, you just want to play on them.”* Children identified a need to limit the amount of time spent on technological devices: *“You could reduce your time down on a tablet or computer so that you’re not always on it.”* This suggests that children are aware of the negative impact that technology has on PA behaviours. 

Prior to data collection, children received a tutorial on how to use all the relevant PA measurement equipment, and were also provided with guidance on how to complete the PA diaries. Despite this, there were still instances where HR data was not collected. This may have been due to the HR monitors moving/slipping whilst being worn, consequently meaning that the HR sensors would not detect the children’s heartbeat. HR monitors may have been worn correctly, but the movement/slipping of the sensors is an acknowledged limitation of their use. To overcome this, the researcher provided additional tutorials on how to tighten HR straps using safety pins, or changed the HR strap for a smaller size. In addition to this, equipment was removed for some activities due to the water-based activities or for health and safety reasons, which previous research has also reported [26]. Therefore, children may have been engaging in MVPA, but it may not have been recorded by the HR equipment. The PA diaries provided an indication of the regularity of these circumstances, and meant that water-based PA could be accounted for. Therefore, future research may wish to consider using a variety of methods, as these can be effectively integrated to ensure all PA is captured.

As indicated by previous literature, GPS data loss may be attributable to problems with the GPS device (e.g., lack of signal, inaccurate positioning, or loss of battery power) and/or children’ handling i.e., forgetting to wear or switch on the device [72]. Within this study, there was a lack of GPS signal reported for time spent indoors due to inconsistent/interrupted satellite connectivity. This meant that it was difficult to explore HR according to this location. Additionally, GPS locations such as ‘home’ and ‘school’ environments may encompass ‘outdoor’ PA opportunities, as opposed to being considered as purely indoor. In the context of this study, boys increased school time compared with girls may be a result of engagement in outdoor school PA, which could further explain KS3 boys’ sedentary behaviour when at home. Future research may wish to develop the home and school environment further, to include ‘home outdoor’, and ‘school outdoor’ within the location index. 

PA diaries are subject to the children’s perception of intensity as well as the accuracy of their memory when completing the diary. Therefore, from the diaries that were collected, there may have been some underestimation or overestimation regarding the duration and intensity of PA participation by the children. However, the objective PA measures used limited the impact of the inaccurate reporting of PA. Children’s abilities to accurately self-report PA has previously been outlined in the literature [73].

Future research exploring PA behaviours should adopt a mixed-methods approach to enhance the context and meaning of the gathered data. Using HR, GPS, PA diaries, or focus groups in isolation would have limited the ‘richness’ of this study. A deeper understanding is provided when combining these measures. In addition to this, the nature of this study was cross-sectional, and details of PA behaviours were only representative of one particular time period within the school academic year. Future research may look to explore PA behaviours longitudinally across the school year.

## 5. Conclusions

The mixed-methods approach to PA data collection this study implemented provides an insightful understanding of children’s PA behaviours by exploring children’s PA locations, PA according to gender, school key stage, and also by exploring barriers and facilitators to children’s PA behaviour. Fifty-two percent of children (boys = 52%, girls = 48%) met the current UK recommended PA guidelines [7] of at least 60 min of MVPA per day. Although not statistically significant, girls took part in more MVPA than boys. Furthermore, KS3 children took part in more MVPA than KS2 children. Therefore, the findings of this study are not comparable and instead contrast with previous literature, in terms of both gender [7,15,45,46] and age differences [18,49,50,51].

Boys and KS3 spend greater time on foot, which includes time on the street and commuting to/from school, with girls in both KS2 and KS3 spending significantly more time in motorised transport than their male counterparts. This supports previously discussed literature regarding girls and younger children [19,20] having less freedom. KS3 children spent significantly more time at home compared with their younger KS2 counterparts. Therefore, girls and KS2 children in the current study were more likely to spend their time in parks and open spaces than boys and KS3 children.

This study reveals that in school versus out of school MVPA levels were similar; however, the school environment showed KS3 children to engage in significantly more MPA than KS2 children, and KS2 boys engaged in more VPA than KS2 girls. These differences could be explained due to school PA clubs being shaped according to KS3 children’s preferences. Therefore, there may be a greater need for schools to acknowledge younger children’s PA preferences, and schools should stage PA clubs that encompass the wider range of abilities. The gender differences in activity at home reveals KS3 boys to engage in significantly more SB than KS3 girls. These behaviours could be partly explained by qualitative data, which highlighted the use of technology by KS3 boys. This highlights how children are aware of the SB associated with technology, particularly in the home environment.

There has been limited research that adopts a mixed-method design incorporating the use of GPS technology, and these measures continue to show promise for exploring PA according to location. Focus groups and PA diaries proved to be important in establishing greater context for PA levels, particularly exploring children’s voice in a deductive approach to follow quantitative data collection. Children took part in PA for enjoyment, socialising with friends/family and also because many of the PA promoting environments are local to them. However, KS2 children reported a greater need for their voices to be heard when deciding on which activities to be staged as part of the school’s extracurricular programme. Barriers such as technology, time, cost of equipment, and poor weather conditions were identified as limiting factors for children’s engagement in PA, which indicates that there is a need to develop programmes that encompass children’s preferences, and consider weather conditions to promote a more encouraging and appealing PA environment. The study extended knowledge of how children’s PA behaviours according to gender and age can be explored using a mixed-methods research design in a school-based sample.

## Figures and Tables

**Table 1:** Breakdown of children according to school key stage (KS2 = 9–11 years; KS3 = 11–13 years) and gender.

Key Stage (and Age Range)	Boys	Girls	Total
2 (School Years 5–6, 9–11 years of age)	42	32	74
3 (School Years 7–8, 11–13 years of age)	15	30	45
**Total**	**57**	**62**	**119**

**Table 2:** Mean daily time and percentage of total time spent in heart rate (HR) intensities of all participants (n = 60), and number of children meeting physical activity (PA) guidelines (n = 31). SB: sedentary behaviour, LPA: light PA, MPA: moderate PA, VPA: vigorous PA, MVPA: moderate–vigorous physical activity.

Variable	SB (min) Percentage SB Time (%)	LPA (min) Percentage LPA Time (%)	MPA (min) Percentage MPA Time (%)	VPA (min) Percentage VPA Time (%)	MVPA (min) Percentage MVPA Time (%)	Meeting PA Guidelines	
						No. Children	Percent of Children (%)
Boys	207.6 (±122.4)	200.9 (±146)	53.9 (±52.9)	14.6 (±24)	68.6 (±63.8)	16	52
	45.7% (±25.4%)	39.6% (±19.8%)	11.7% (±10.2%)	2.8% (±3.5%)	13.8% (±11.2%)		
Girls	204.4 (±138.8)	172.9 (±143.1)	91.9 (±103.6)	15 (±30.3)	106.9 (±117.5)	15	48
	45.3% (±28.9%)	33.6% (±23%)	17.9% (±19.5%)	3.1% (±6.4%)	20.9% (±22%)		
KS2 (9–11 years)	216 (±143)	183.1 (±145.4)	64 (±62.4)	13.2 (±18.2)	77.2 (±72.7)	18	58
	46.2% (±27.3%)	36.5% (±23%)	14% (±12.5%)	2.9% (±4.5%)	16.5% (±14.6%)		
KS3 (11–13 years)	191.8 (±110.6)	191 (±144.7)	87 (±108.4)	17.1 (±36.6)	104 (±122.5)	13	42
	44.4% (±27.2%)	36.4% (±20%)	16.2% (±20%)	3% (±6)	18.8% (±21.9%)		
KS2 Boys	183.6 (±119.6)	204.1 (±143.3)	59.3 (±53.8)	13.3 (±14.2)	72.6 (±63.5)	11	35
	40.5 (±24.1)	42.6 (±20.1)	13.6 (±10.9)	2.8 (±2.8)	15.7% (±12.1%)		
KS2 Girls	259.2 (±163.6)	155.2 (±148.3)	70.1 (±73.8)	13.1 (±23)	83.3 (±85.5)	7	23
	53.9 (±30.1)	28.4 (±24.4)	14.4 (±14.7)	3.1 (±6.2)	17.6% (±17.8%)		
KS3 Boys	260.9 (±117.5) *	193.7 (±160.3)	41.9 (±51.6)	17.6 (±39)	59.5 (±67.3)	5	16
	57.1 (±25.6)	32.9 (±18.2)	7.3 (±7.4)	2.7 (±4.8)	9.6% (±8.5%)		
KS3 Girls	153 (±87.9) *	189.5 (±140.7)	112.3 (±124.4)	16.8 (±36.5)	129 (±140.5)	8	26
	37.2 (±26)	38.4 (±21.3)	21.2 (±23.1)	3.2 (±6.8)	24% (±25.6%)		
Overall	205.9 (±130)	186.4 (±143.9)	73.5 (±84.6)	14.8 (±27.2)	88.4 (±96.6)	31	100
	45.5% (±27%)	36.5% (±21.6%)	14.9% (±15.9%)	2.9% (±5.2%)	17.5% (±17.9%)		

* Statistically significant difference between gender, key stage and gender within key stage (*p <* 0.05).

**Table 3:** Mean percentage of time (±SD) in each location according to gender, key stage, and gender within each key stage.

Variable	Home	School	Other Indoor Location	Motorised Transport	On Foot	Outdoors (Not Including ‘On Foot’)	Outside (Combining ‘On Foot’ and ‘Outdoors’)
Boys	30.8 (±23.1)	41.4 (±22.7) **	1.9 (±3.8)	8.3 (±12.4) *	4.8 (5.8)	12.7 (±11.8)	15.5 (±10.8)
Girls	32.7 (±7.5)	23.4 (±26.4) **	4.1 (±8.9)	17.7 (±17.6) *	4.3 (5.8)	17.8 (±14.8)	17.4 (±13.2)
KS2 (9–11 years)	24.8 (±21.2) *	32.6 (±25)	3.2 (±8.4)	16.6 (±18.4) *	3.7 (±4.9)	19.1 (±12.7) **	19.4 (±13.2) *
KS3 (11–13 years)	41.6 (±27.7) *	31.4 (±28)	2.9 (±4.4)	8.4 (±10) *	5.7 (±6.8)	10 (±13) **	12.4 (±8.8) *
KS2 Boys	26.4 (±21.9)	40.2 (±23.8) *	2.1 (±4.2)	11.3 (±13.9) *	3.2 (±3.8)	16.8 (±10.9)	17.3 (±11.3)
KS2 Girls	22.7 (±20.9)	22.5 (±23.8) *	4.6 (±12)	23.7 (±21.6) *	4.3 (±6)	22.2 (±14.6)	22.3 (±15.4)
KS3 Boys	40.5 (±24)	44.3 (±21.2)	1.4 (±2.7)	1.7 (±2.4) *	8.3 (±8.1)	3.7 (±8.5)	11.5 (±9)
KS3 Girls	42.2 (±30.3)	24.2 (±29.4)	3.7 (±4.9)	12 (±10.8) *	4.3 (±5.7)	13.6 (±14.1)	12.8 (±8.9)
Overall	31.8 (±25.3)	32.1 (±26.1)	3.1 (±7)	13.2 (±15.9)	4.5 (±5.8)	15.3 (±13.5)	16.5 (±12)

* Statistically significant (two-tailed) difference between gender, key stage, and gender within key stage (*p* < 0.05); ** Statistically significant (two-tailed) difference between gender, key stage, and gender within key stage (*p* < 0.01). Key stage (*p* < 0.01).

**Table 4:** A summary of themes and examples of raw data extracts for barriers to and facilitators for PA.

Themes	Selected Quotes From Children	
	Barriers to PA	Facilitators for PA
**Individual**		
Time	*“If I have too much homework, exams and stuff.”*	*“… however long you do on a physical activity, you get half that time on technology* *or something like that”*
Fitness/Health	*“If you’re ill”*	*“I don’t want to be all weaker when I’m older, I want to stay healthy”*
Fun and enjoyment	*“They might not like it, so they might not want to do it.”*	*“I do it because I like dancing and it just makes me happy”*
Equipment cost	*“… if they don’t have like the right equipment to do it.”*	*“Maybe lower the price a little bit so that people don’t have to wait loads to save up”*
**Social Environment**		
Friends and family	*“Might have to look after their family and it might stops [sic]* *you having their social time with their friends and going out”*	*“I do runs with my dad, I go on my bike”*
Socialise	*“… sometimes some of the people might have other plans”*	*“It’s a chance to meet up with friends”*
**Physical Environment**		
Location/Environment	*“Not exercising and getting outside.”*	*“I go up to the college car park because it’s big and loads of my friends just play there.”*
Club	*“… have to be good at it to go there and stay there”*	*“… because that’s where the club [is], it’s what I do and I’m a part of that team”*
Facilities	*“That’s where you play, that’s the home ground or your home team. You have to go there.”*	*“There’s trampolining at, I can’t remember the school now but it’s a high school.”*
**Policy**		
Rewards	*“…make it into like a fun game and whoever did the most exercise in that period of time would get a prize or something like that.”*	*“… in periods of time when who does the most exercise you win a prize, so you’re kind of pushing the students to work for the prize.”*
Promotion	*-*	*“… put a word on Facebook and tell people what’s happening and let everyone know happening and try and bring some people down to it.”*
Child Voice	*“People might not like what the variety is”*	*“You can ask what they like the most, and start clubs and get them to come.”*
Awareness	*-*	*“Like do a survey to see what they would want to do instead of like saying we’re going to do hockey today because some people might not like it.”*
**Other**		
Weather	*“… if it’s in the winter, some people don’t have motivation because it’s quite cold and dark, and if it’s muddy.”*	*“… you need the activity, the exercise and the nice fresh air, instead of being stuck indoors, stuff like that.”*
Technology	*“When you’ve got a phone or like a console, you don’t think as much about getting active, you just want to play on them.”*	*“You could reduce your time down on a tablet or computer so that you’re not always on it.”*
